# Systematic evaluation of implementation fidelity of complex interventions in health and social care

**DOI:** 10.1186/1748-5908-5-67

**Published:** 2010-09-03

**Authors:** Henna Hasson

**Affiliations:** 1 Lund University School of Economics and Management, Department of Business Administration, 220 07 Lund, Sweden; 2 Vårdal Institute, Swedish Institute for Health Sciences, 221 00 Lund, Sweden; 3 Karolinska Institutet, Department of Learning, Informatics, Management and Ethics, Medical Management Centre (MMC), 171 77 Stockholm, Sweden

## Abstract

**Background:**

Evaluation of an implementation process and its fidelity can give insight into the 'black box' of interventions. However, a lack of standardized methods for studying fidelity and implementation process have been reported, which might be one reason for the fact that few prior studies in the field of health service research have systematically evaluated interventions' implementation processes.

The aim of this project is to systematically evaluate implementation fidelity and possible factors influencing fidelity of complex interventions in health and social care.

**Methods:**

A modified version of The Conceptual Framework for Implementation Fidelity will be used as a conceptual model for the evaluation. The modification implies two additional moderating factors: context and recruitment. A systematic evaluation process was developed. Multiple case study method is used to investigate implementation of three complex health service interventions. Each case will be investigated in depth and longitudinally, using both quantitative and qualitative methods.

**Discussion:**

This study is the first attempt to empirically test The Conceptual Framework for Implementation Fidelity. The study can highlight mechanism and factors of importance when implementing complex interventions. Especially the role of the moderating factors on implementation fidelity can be clarified.

**Trial Registration:**

Supported Employment, SE, among people with severe mental illness -- a randomized controlled trial: NCT00960024.

## Background

Health service interventions are often highly complex, compared to efforts like drug trials or trials of surgical procedures [1]. Health service interventions consist of a number of components that might act both independently and inter-dependently. This can challenge the evaluation of the program impact [2]. Experimental studies that most often are used to evaluate interventions give little information on why certain effects were or were not found. In addition, no information is gained on whether complex interventions were implemented as intended [3]. Thus, there might be a risk in evaluating a program that was described but not implemented [4]. For instance, a classical study by Dobson and Cook [4] regarding a program for ex-offenders found that only one in twenty consumers actually received the program as described in the methods section. Thus, the outcome data could not be attributed to the program as described. Other studies examining programs to help people with mental illnesses obtain employment found that program outcomes among the intervention groups were weakest for those in poorly implemented programs [5].

As a result, it has been recognized that aside from outcome evaluations, it is necessary to gain insight into the 'black box' of interventions [6]. It has been suggested that a process evaluation including information about program implementation is needed to evaluate complex interventions [3,7]. A study of intervention implementation process could improve the validity of intervention findings [6,8,9] and help to explain for what specific reasons an intervention succeeded or failed [4]. For instance, studies with a type III error, *i.e*., a failure to implement a program as planned, could erroneously conclude that lack of program impact was due to attributes of the particular intervention if no process measures were collected [4]. Process evaluation can also allow better judgment of transferability of potentially effective programs to other settings [1]. However, several literature reviews of intervention research have concluded that few prior studies in the field of health service research have systematically documented implementation processes of intervention programs [7,9,10].

In general, a process evaluation examines what the program is and how it is delivered to the target clients [11]. Implementation fidelity has been used as a measure for the degree to which an intervention was implemented as was intended [12]. These concepts overlap [6], but the basic idea of both concepts is to compare the program itself and its actual delivery to a standard of the program that describes the intended program and its intended implementation [11]. Several aspects of the program delivery can be measured. Steckler *et al. *[13] defined key components to be included in a process evaluation. They suggested that a systematic evaluation should be done of the procedures that were used to attract participants (recruitment), the proportion of intended clients who actually participated in the program (reach), the extent to which participants engaged in the activities of the program (dose received), the amount of intended components of the program that were delivered (dose delivered), the extent to which the program was implemented as planned (fidelity), and the aspects of the context, (*i.e*., larger social, political, and economic environment that may have influenced implementation). Other studies have focused more precisely on the concept of implementation fidelity and suggested that it can be defined in terms of five elements regarding to what extent the delivered intervention correspond to the designed intervention (adherence), amount of an intervention received by participants (exposure or dose), quality of the program delivery, participants' engagement in the program activities (participant responsiveness), and presence or absence of the critical program elements (program differentiation) [10,12]. Several authors have suggested that multiple components need to be measured in order to achieve a comprehensive picture of implementation processes and fidelity [9,12,13]. However, several literature reviews of implementation fidelity concluded that many prior process studies have included too few components when analyzing implementation fidelity [10,12]. Most of these studies have only evaluated adherence, the extent to which the delivered interventions correspond to the designed intervention [12], which alone does not give understanding of factors affecting implementation [9]. It has been stated that a more standardized methodology for studying fidelity is needed [12]. Thus, there is a need for more systematic implementation evaluations that measure several aspects of an implementation process.

### The conceptual framework

**Figure 1:**
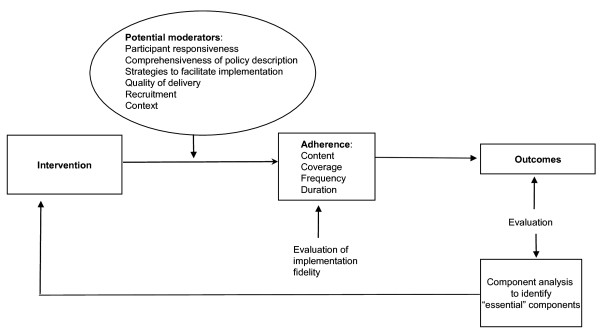
**The modified conceptual framework for implementation fidelity (originally from Carroll et al**.).

Carroll *et al. *[9] proposed a framework for evaluation of implementation fidelity, which currently is the most complete conceptual framework for implementation fidelity. The framework includes components of implementation fidelity and factors that may influence the degree of fidelity, referred to as moderating factors. The measurement of implementation fidelity is a measurement of adherence, with its subcategories -- content, frequency, duration, and coverage (dose). Thus, adherence relates to the content and dose of the intervention, *i.e*., whether the active ingredients of the intervention have been received by the participants as often and for as long as was planned. Intervention complexity, facilitation strategies, quality of delivery, and participant responsiveness were included in the framework as moderating factors. Intervention complexity has been found to influence the implementation fidelity, *i.e*., complex interventions were more difficult to implement with high fidelity than simple interventions [14]. Complexity refers to both description of the intervention and the real nature of the intervention. Interventions described in detail are more likely expected to be implemented with high fidelity than ones with vague descriptions. Facilitation strategies, such as provision of manuals, guidelines, training, and feedback, may be used both to optimize and to standardize implementation fidelity. However, more facilitation strategies do not necessarily mean better implementation. Instead facilitation might be highly dependent on the complexity of the intervention. Quality of delivery concerns the appropriateness of the delivery process for achieving what was intended. Dusenbury *et al. *[12] defined quality of delivery as 'the extent to which a provider approaches a theoretical ideal in terms of delivering program content.' Participant responsiveness refers both to individuals receiving the intervention and individuals responsible for delivering it. Higher levels of implementation fidelity are assumed to be achieved if those responsible for delivering an intervention are enthusiastic about it. Similarly, the uptake of the intervention depends on the responsiveness of those receiving it. The authors of the framework suggest that the moderators have complex relationships to each other and to the implementation fidelity. For example, facilitation strategies may improve quality of delivery, which in turn may influence participants' commitment to the intervention. However, the potential moderating effect of intervention complexity makes that impact more complicated. The authors suggest that there may be interaction effects between moderators, *i.e*., when the effect of one factor is dependent on the level of another. In summary, the framework suggested that different moderating factors might affect, positively or negatively, the implementation process and its fidelity. These factors interact with each other and the effect of one factor on fidelity might be influenced by another moderating factor. The framework suggested that all these factors should be evaluated systematically when conducting a process evaluation. In this project, two additional moderating factors were included in the framework, namely context and recruitment. The modified framework is presented in Figure 1. Importance of context for program implementation has been highlighted by several other authors [3,15-17]. Lipsey [3] emphasized the importance of taking into account surrounding social systems, such as structures and cultures of organizations and groups, inter-organizational linkages, and historical as well as concurrent events, when assessing program implementation. Pettigrew and Whipp's [15] model of strategic change management defined context together with content and process as main determinants of change. Recruitment refers to procedures that were used to attract potential program participants. Baranowski and Stables [18] argued that recruitment was a key process evaluation component. Some of the aspects to be evaluated were suggested to be reasons for nonparticipation among potential participants, subgroups that were less likely to participate, and consistency of recruitment procedures among potential participants. Steckler *et al. *[13] argued that an evaluation of recruitment can contribute to correct generalization of findings, *i.e*., not generalization results for subgroups that have chosen not to participate. In this study, the modified framework will be used as a conceptual model to structure the data collection and analyses to identify mechanism and factors that might influence the implementation of complex interventions.

The aim of this project is to evaluate systematically implementation fidelity and possible factors influencing fidelity of complex interventions in health and social care. The purpose is to test the conceptual framework for implementation fidelity proposed by Carroll *et al. *and contribute to the knowledge base of how implementation fidelity and moderating factors can be evaluated for complex interventions.

## Methods

### Design of the study

The multiple case study method will be used to investigate the implementation processes of three intervention studies. Thus, a case is defined as an intervention study. Each case will be investigated in depth and longitudinally, using both quantitative and qualitative methods. The case study method has been proposed to be a suitable method for conducting longitudinal research of change processes [17,19].

### Descriptions of the cases, i.e., interventions

All three interventions are complex in nature, including several active ingredients. In addition, the interventions are conducted in complex health or social care environments where several professional groups or care provider organizations cooperate. The three interventions are briefly described below.

### Continuum of care for frail elderly persons, from the emergency ward to living at home

The intervention involves development, implementation, and evaluation of an integrated care chain for frail elderly people. The care chain will cover the older person's contacts with community care providers (home help services, home nursing, and rehabilitation), primary care, the hospital emergency department and hospital ward. A case manager and a multi-professional team will help the elderly people and their relatives to coordinate care contacts. The theoretical framework for the study is that integrated care with a case manager creates networks of resources and services over time and between different healthcare providers, particularly between health and social care. This is expected to improve health-related quality of life, increase satisfaction with care and rehabilitation, decrease older persons' emergency care consumption, and influence cost efficiency at the community level. The study design is a randomized controlled study with a total of 200 participants divided into intervention and control group. The study is conducted in the city of Mölndal in western Sweden. The possible effects of the intervention on participants' capability to perform activities, health-related quality of life, satisfaction with care, and emergency care consumption will be evaluated at three, six, twelve and twenty-four months after the baseline measurement. The responsible researchers are Synneve Dahlin-Ivanoff, PhD (Medicine), Professor and Katarina Wilhelmson, PhD (Medicine) at the Sahlgrenska Academy at Göteborg University.

### Palliative care in community nursing homes for older people -- support for nursing staff

The intervention involves development, implementation, and evaluation of a support program in palliative care for nursing staff and workplace leaders working in community nursing facilities for older people. The support program is based on a study circle model that combines participants' reflections and discussions based on their expertise and work experiences. The model includes having participants learn to question their work practices and develop new ways of working and solving problems at work. The program consists of regular meetings for each professional group and cross-professional workshops. Reading materials regarding palliative care and other related relevant subjects will be provided prior to a study circle. An external facilitator will lead the circles. Participants are expected to get direct support in terms of improved knowledge as well as indirect support in terms of a collective platform for reflections, discussion, stimulation, and concrete improvement work. The theoretical framework for the study is that support in terms of expertise development and coaching positively affects staff attitudes towards care recipients, their work satisfaction, and general wellbeing. The intervention is conducted in a quasi-experimental design in six nursing homes in the city of Malmö in southern Sweden. The possible effects of the intervention on staff satisfaction, work climate, and general well-being are evaluated at six and eighteen months after the baseline measurement. The responsible researcher is Anna-Karin Edberg, PhD (Medicine), Professor, Lund University.

### Supported employment among people with severe mental illness -- a randomized controlled trial

The study involves implementation and evaluation of an evidence-based method, supported employment (SE), for vocational rehabilitation for people with mental illnesses. The purpose of SE, according to the Individual Placement and Support model, is to help people with severe mental illness find and keep employment in a competitive market. SE has been widely recognized as the most effective approach to increasing work opportunities for people with severe mental illness [20-22], but has not been tested in a Swedish welfare context. This is the first randomized controlled SE study in Sweden. The theoretical framework for the study is that work enables people to integrate socially and provides them with opportunities to explore and master their environment and thereby become integrated in society. The study is conducted in the city of Malmö in southern Sweden. The possible effects of the intervention on clients' vocational outcomes, such as employment rate and monthly income, as well as on their non-vocational outcomes, such as quality of life and sense of empowerment, will be evaluated at six and eighteen months after the baseline measurement. The responsible researcher is Ulrika Beijerholm, PhD (Medicine), Lund University.

### The research team

The author of the paper is the principle investigator of this implementation project. The project is a part of larger research programs at the Vårdal Institute, where the three above-mentioned intervention projects are also being conducted. The responsible researchers mentioned above are responsible for designing, conducting, and evaluating the impact of the intervention studies. Thus, the investigation of implementation process and its fidelity is conducted by other researchers than those involved in the development of the interventions. However, some data collection is conducted in collaboration so that participants and other stakeholders need not experience excessive burdens with interviews, questionnaires, and observations.

### The evaluation plan

An overall process evaluation plan was developed for this project. This is presented in Table 1. In addition, more detailed evaluation plans for each intervention study were created (see additional files 1, 2 and 3). The modified framework for implementation fidelity was used to define the areas to be measured. These are presented at the first column of Table 1. Steckler *et al*.'s [13] stepwise approach to designing a process evaluation was used as a tool for planning the practical steps in the evaluation process. In accordance with the approach, first a description of the actual program and its theoretical basis, purpose, core inputs, and expected outcomes was made. This description was summarized in a logic model. Separate logic models were created for each of the three intervention studies. As an example, the logic model for the Continuum of care for frail elderly persons, from the emergency ward to living at home intervention project is presented in Table 1.

**Table 1:** The logic model of *Continuum of care for frail elderly persons, from the emergency ward to living at home *intervention

Core inputs	Immediate Impacts	Short-Term Impacts	Impacts	Health Outcomes
Geriatric assessment at emergency department,	Contact between emergency department and community case manager,	Community care will have increased information regarding the needs of the older person, increased contact between emergency healthcare and community social care,	Possibilities for earlier discovery of problems, earlier care and rehabilitation efforts and changes in care and rehabilitation plans, better uptake of older people's viewpoints	Maintained functional ability, increased life satisfaction, reduced number of visits to the emergency department,
Case manager and multi-professional team at the community care,	Case manager has early contact with older person at hospital, continuous contact between case manager and older people, early contact with older peoples' families			Reduced number of stays in hospital wards, higher satisfaction with community care and rehabilitation
Care planning after hospital discharge at older person's home		Older people will have more knowledge of whom to contact when they need help, increased participation opportunities for older people and their families in care planning		

In the second step, a detailed description of the components of the programs was created. At this stage, each component of the intervention and its intended delivery was described as these were stated in a program plan. Also, the content and delivery of the program for the control group was described. Amount of intervention services and frequency of delivering these services were described in detail. Table 2 presents the delivery process of the Continuum of care for frail elderly persons, from the emergency ward to living at home intervention.

**Table 2:** Planned delivery of the *Continuum of care for frail elderly persons, from the emergency ward to living at home *Intervention

INTERVENTION GROUP	
**Emergency department**	A nurse with geriatric expertise makes an assessment of the elderly patients' needs of rehabilitation, nursing, and geriatric care.
	
	For participants who are admitted to the hospital ward, the geriatric assessment is transferred to the ward nurses.
	
	The case manager and the multi-professional team in the community are informed that the patient has visited the emergency care, and whether he/she was transferred to a hospital ward or returned home.
	
	The geriatric assessment is sent to the case manager and the multi-professional team in the municipality.

**Hospital ward**	The community case manager is responsible for contacting the ward and the elderly person.
	
	The case manager visits participants in the ward, if necessary, contacts the participants' relatives, and initiates support for relatives if necessary.
	
	The case manager continues to have contact with the hospital ward so that discharge planning can start early.
	
	Discharge planning is done in collaboration between the case manager, a qualified social worker, the patient, as well as the nurse and physician in charge at the ward.

**Community care**	The case manager contacts participants returning home after visiting the emergency department and offers care planning. She also initiates support for patients' relatives if necessary.
	
	The case manager and the multi-professional team make a care plan a couple of days after discharge from the hospital ward. Care planning is done at the older person's own home instead of in the hospital ward, which is the traditional model.
	
	The care plan is based on the results in the geriatric assessment made at the emergency department. Further assessment is made regarding patients' functional abilities, health status, diseases, and ongoing and planned treatment and care. All planning is done in consultation with the patient.
	
	The multi-professional team informs other professionals and care providers, such as home help services and home nursing care, regarding the plan made.
	
	The case manager follows up the care plan within a week, via telephone or home visit, to ensure that everything is working and no new problems have arisen.
	
	The participants are advised that the case manager is available for questions, problem solving, and assistance during office hours.
	
	The case manager has telephone contact with participants once a month except in cases where more frequent contact is needed.

**Primary care**	Patient's general practitioner is informed by letter that the individual is participating in the research project. Information is given regarding content of the project, *i.e.*, the role of the case manager, and her contact information.

The control group receives traditional care that differs from the intervention in the following aspects:	

**CONTROL GROUP**	No nurse with geriatric expertise available at the emergency department, which implies that no geriatric assessment is made.
	
	No case manager or multi-professional team available, which implies among other things that the community is not informed if an older person has visited emergency department. Nor is the community informed when older people have been hospitalized in a ward if these people do not have community home help services or nursing care. It implies also that the elderly people do not have a one single contact person; instead they contact different care organizations when needed.
	
	For patients being hospitalized, a care plan is made at the hospital ward by the community social worker, community nurse, and rehabilitation staff when necessary.
	
	Follow-up of the care plan is done at patient's home by care providers, *i.e.*, home help providers or home nursing providers.
	
	No follow-up for individuals who don't receive home help or home nursing.

In the third stage, general process questions were developed (second column in Table 3). One to three questions were developed for each fidelity component and potential moderating factor. For instance, subcategories of fidelity are measured through questions: 'Was each of the intervention components implemented as planned?,' 'Were the intervention components implemented as often and for as long as planned?' and 'What proportion of the target group participated in the intervention?.' To measure participant responsiveness, three questions were developed: 'How did the participants become engaged in the intervention services?,' 'How satisfied were the participants with the intervention services?' and 'How did the participants perceive the outcomes and relevance of the intervention?.' After developing the general process questions, more specific questions for each of the intervention projects were developed. These are described in additional files 1, 2 and 3.

**Table 3:** The general evaluation plan including areas to measure, general process questions, data sources and data collection methods


**Areas to measure**	**General process questions**	**Data source and data collection method**

**Evaluation of adherence**		

Content	Was each of the intervention components implemented as planned?	- observations of work practices - project leaders' logbooks - interviews with stakeholders and participants - Intervention 3: a previously developed and validated instrument IPS-fidelity scale will be used

Frequency/Duration (Dosage, Dose delivery)	Were the intervention components implemented as often and for as long as planned?	- observations of work practices - project leaders logbooks - interviews with stakeholders and participants

Coverage (Reach)	What proportion of target group participated in the intervention?	- interviews with project leaders and other relevant stakeholders

**Potential moderating factors**		

Participant responsiveness (Dose received)	How were the participants engaged with the intervention services? How satisfied were the participants with the intervention services? How did the participants perceive the outcomes and relevance of the intervention?	- project leaders' logbooks - interviews with project leaders and participants - questionnaire items to participants included in the follow-up measurements

Intervention complexity	How complex is the intervention?	- a group of external researchers will evaluate the intervention complexity

Comprehensiveness of policy description	How specific is the interventions description?	- a group of external researchers will evaluate the comprehensiveness of policy description

Strategies to facilitate implementation	What strategies were used to support implementation? How were these strategies perceived by staff involved in the project?	- interviews with project leaders, participants and other relevant stakeholders, - questionnaire items to participants included in the follow-up measurements

Quality of delivery	How was the quality of delivering the intervention components?	- interviews with project leaders and participants, - observations of work practices

Recruitment	What recruitment procedures were used to attract individuals to the intervention? What constituted barriers to maintaining involvement of individuals?	- interviews with those who recruited the participants - interviews with project leaders and participants

Context	What factors at political, economical, organizational, and work group levels affected the implementation?	- interviews with project leaders, participants, and other relevant stakeholders - project leaders' logbooks - questionnaire items to participants included in the follow-up measurements

### Data collection and analyses

Data will be collected for each of the three intervention studies during the entire intervention period. A multi-method approach will be used. Data collection methods include key informant interviews, non-participant observations, questionnaire studies, analysis of participants' logbooks, and other document analysis. The data collection methods for answering each of the process questions are described in the third column in Table 3. For instance, observations of work practices, project leaders' logbooks and interviews with project leaders, participants, and other relevant stakeholders will be used to answer the questions concerning implementation fidelity. In addition, in the third intervention a previously developed and validated instrument, Supported Employment Fidelity Scale [23], is used to evaluate implementation fidelity. To evaluate adequacy of strategies to facilitate the implementation, process interviews with the relevant stakeholder will be conducted and questionnaire items will be added in follow-up questionnaires to intervention participants. To measure the complexity of the intervention programs, a group of external researchers will be used. Contextual factors will be measured with interviews, logbooks, and questionnaire items. For instance, in the Palliative Care In Community Older People Nursing Homes -- Support For Nursing Staff project, a previously validated questionnaire, the Dimensions of the Learning Organization Questionnaire [24,25], will be translated to Swedish and used to measure participants' perceptions of learning culture in their organizations. More detailed descriptions of data collection methods for each of the intervention studies are presented in additional files 1, 2 and 3.

Some data collection such as interviews and observations will be conducted in collaboration with other researchers focusing on process aspects at the Vårdal Institute. This is done to minimize the load for respondents and to best use the resources of the researchers.

Content analysis of the qualitative data, *i.e*., logbooks, interviews, observations, and document material will be conducted. As has been suggested [26], a coding scheme will be created and tested prior to the analyses. Results from the questionnaire surveys will be analyzed with both descriptive and analytical methods. Results of the surveys enable analyses regarding variations within the intervention group and its possible relationship with outcome variables. For instance, users' participation in the program can be studied in relation to their results in the follow-up outcome measurements.

### Ethical approval

Data collection in this project was included in the ethical applications of the intervention projects. Ethical approvals have been granted for the first intervention study: (Gothenburg University dossier number 413-08) and the third intervention study (Lund University dossier number 202/2008). For the second study, an ethical application was sent to the research ethics committee at Lund University. The committee reported that they didn't identify any ethical hinders for conducting the study (dossier number 2009-527), but made a decision that in accordance with legislation no formal ethical approval was needed for the study.

## Discussion

The aim of this project is to systematically evaluate implementation fidelity and possible factors influencing fidelity of complex interventions in health and social care. The intention is to empirically test the conceptual framework for implementation fidelity proposed by Carroll *et al. *[9]. The framework was modified in such a way that two additional moderating factors, context and recruitment, were included in the framework. The purpose is to contribute to the knowledge base on development of systematic evaluation of implementation of complex interventions. This will highlight the mechanism and factors of importance when implementing complex interventions. Especially the role of the moderating factors influencing implementation will be clarified.

The study also presents a practical example of how to develop a systematic process evaluation for complex interventions. The results of this study can be used to interpret the results of the outcome evaluation of the interventions. Information will be gained on how, when, and in what context the interventions work. This information can be used for practical future program planning.

Some practical issues relevant to the conducting of this study will be briefly discussed. First, these interventions are conducted in local practices, but in a research context. Programs implemented as part of research projects usually receive considerable support to achieve high fidelity [12]. Outside of research context, implementation usually takes place in less ideal circumstances [10]. Thus, it is possible that the factors affecting implementation and its fidelity in this project are not totally comparable to real-life situations. Nonetheless, as Dane and Schneider [10] point out, understanding fidelity under the research conditions is crucial for a field of practice to advance. The next step would be to study the implementation of these programs after the research program.

The three intervention studies included in the project represent different type of health service interventions. This will offer an opportunity for cross-case comparison of different interventions. Knowledge will be gained regarding each type of intervention specific, and more general knowledge will be obtained when comparing the cases. The strength of using a case study design lies in the opportunity to collect multiple types of data, enabling development of a comprehensive, in-depth picture of the implementation processes.

A process evaluation often requires a large amount of data collection, which makes it time-consuming and expensive. This project offers an example of how collaboration between different researchers within a large project can enable collection of process data. The collaboration with data collection enables a rich data material. For instance, stakeholder interviews and worksite observations will be conducted in collaboration with other researchers. In addition, questions regarding participants' experiences of the program implementation will be included in follow-up outcome evaluation questionnaires instead of conducting separate questionnaire surveys or participant interviews. Using results of interviews that have been conducted by other researchers might have some limitations, such as not obtaining primary source information. On the other hand, the positive factors concerning time, resources, and respondent burden were considered to carry more weight.

In any type of process analysis, a choice has to be made regarding what data should be collected. On one hand, several implementation process components need to be measured to be able to understand the process [9]. On the other hand, for practical reasons a selection of collected data needs to be made. The study presents a systematic way of evaluating implementation fidelity and factors potentially affecting fidelity. However, the study does not cover all potential factors influencing implementation of complex interventions. This is an attempt to measure the most essential components identified in the prior studies. The goal is that this study can contribute to knowledge of what factors should be included in future process evaluations.

## Competing interests

The author declares that they have no competing interests.

## Supplementary Material

Additional file 1 **A process-evaluation plan for the Continuum of care for frail elderly persons, from the emergency ward to living at home**. Click here for file

Additional file 2 **A process-evaluation plan for Palliative care in community older people nursing homes - support for nursing staff**. Click here for file

Additional file 3 **A process-evaluation plan for the Supported Employment (SE) among people with severe mental illness - a randomized controlled trial**. the files include detailed evaluation plans for each intervention study. Click here for file
