# Supplementary material for: Systematic evaluation of implementation fidelity of complex interventions in health and social care
Source: Implement Sci. 2010 Sep 3;5:67. doi: 10.1186/1748-5908-5-67 (PMC2942793; doi:10.1186/1748-5908-5-67)
Supplement: Additional file 1 — A process-evaluation plan for the Continuum of care for frail elderly persons, from the emergency ward to living at home. [file 1748-5908-5-67-S1.DOC]

**A process-evaluation plan for the *Continuum of care for frail elderly persons, from the emergency ward to living at home* intervention**

**Program description and logic model**

The aim of the project is to create a chain of care for frail elderly persons from the emergency department, through the ward, and to the elderly person’s home in the community. The intervention is a collaboration between a nurse with geriatric expertise situated at the emergency department, the hospital ward staff, and a multi-professional team for the care of elderly with a case manager in the community. The multi-professional team includes a nurse (the case manager), a qualified social worker, an occupational therapist, and a physiotherapist. The case manager and the team are available to the participants for questions, problem solving, and assistance.

The theoretical framework for the study is that integrated care with a case manager creates networks of resources and services over time and between different health care providers, particularly between health and social care. The idea is also to strengthen the self-reliance of the elderly and their relatives. Effective components are expected to be a geriatric assessment in the emergency department, a community multi-professional team with a case manager, and a care plan made in the older people’s home instead of in the hospital ward. The idea is to involve the older person in the decision-making regarding their care planning and rehabilitation. The older persons find it easier to be involved in that process when they are in their own home environments rather than in a hospital. Another benefit of care planning at home can be that the multi-professional team can directly observe how the older persons manage their activities in their own home environments. Early support will be offered to the relatives and they will be given an opportunity to be involved in decisions and planning of the elderly care.

Table 1. Logic model

| **Core inputs** | **Immediate Impacts** | **Short-Term Impacts** | **Impacts** | **Health Outcomes** |
| --- | --- | --- | --- | --- |
| geriatric assessment at emergency department,  case manager and multi-professional team at the community care,  care planning after hospital discharge at older person’s home | contact between emergency department and community case manager,  case manager has early contact with older person at hospital, early contact with older peoples’ families, continuous contact between case manager and older people | community care will have increased information regarding the needs of the older person, increased contact between emergency health care and community social care,  older people will have more knowledge of whom to contact when they need help, increased participation opportunities for older people and their families in care planning | possibilities for earlier discovery of problems, earlier care and rehabilitation efforts and changes in care and rehabilitation plans, better uptake of older people’s viewpoints | maintained functional ability, increased life satisfaction, reduced number of visits to the emergency department,  reduced number of stays in hospital wards, higher satisfaction with community care and rehabilitation |

**Complete and acceptable delivery**

The ideally implemented care pathway program will consist of four essential care environments: the emergency department, the hospital ward, community care (including home help services, home nursing and rehabilitation), and primary care. The participants are recruited at the emergency department in accordance with the inclusion criteria and randomized to the intervention or control group.

The following steps are taken in a complete and acceptable delivery of the intervention program:

**Emergency department**

- A nurse with geriatric expertise makes an assessment of the elderly patients’ needs of rehabilitation, nursing, and geriatric care.
- For participants who are admitted to the hospital ward, the geriatric assessment is transferred to the ward nurses.
- The case manager and the multi-professional team in the community are informed that the patient has visited the emergency care, and whether he/she was transferred to a hospital ward or returned home.
- The geriatric assessment is send to the case manager and the multi-professional team in the municipality.

**Hospital ward**

- The community case manager is responsible for contacting the ward and the elderly person.
- The case manager visits participants in the ward, if necessary, contacts the participants’ relatives, and initiates support for relatives if necessary.
- The case manager continues to have contact with the hospital ward so that discharge planning can start early.
- Discharge planning is done in collaboration between the case manager, a qualified social worker, the patient, as well as the nurse and physician in charge at the ward.

**Community care**

- The case manager contacts participants returning home after visiting the emergency department and offers care planning. She also initiates support for patients’ relatives if necessary.
- The case manager and the multi-professional team make a care plan a couple of days after discharge from the hospital ward. Care planning is done at the older person’s own home instead of in the hospital ward, which is the traditional model.
- The care plan is based on the results in the geriatric assessment made at the emergency department. Further assessment is made regarding patients’ functional abilities, health status, diseases, and ongoing and planned treatment and care. All planning is done in consultation with the patient.
- The multi-professional team informs other professionals and care providers, such as home help services and home nursing care, regarding the plan made.
- The case manager follows up the care plan within a week, via telephone or home visit, to ensure that everything is working and no new problems have arisen.
- The participants are advised that the case manager is available for questions, problem solving, and assistance during office hours.
- The case manager has telephone contact with participants once a month except in cases where more frequent contact is needed.

**Primary care**

- Patient’s general practitioner is informed by letter that the individual is participating in the research project. Information is given regarding content of the project, i.e. the role of the case manager, and her contact information.

The control group receives traditional care that differs from the intervention in the following aspects:

- No nurse with geriatric expertise available at the emergency department, which implies that no geriatric assessment is made.
- No case manager or multi-professional team available, which implies among other things that the community is not informed if an older person has visited emergency department. Nor is the community informed when older people have been hospitalized in a ward if these people do not have community home help services or nursing care. It implies also that the elderly people do not have a one single contact person; instead they contact different care organizations when needed.
- For patients being hospitalized, a care plan is made at the hospital ward by the community social worker, community nurse, and rehabilitation staff when necessary.
- Follow-up of the care plan is done at patient’s home by care providers, i.e. home help providers or home nursing providers.
- No follow-up for individuals who don’t receive home help or home nursing.

**Process-evaluation questions and data collection methods for answering the questions**

Table 2 below reports questions for the process evaluation and the methods for answering the questions.

| **Areas to measure**  **1.** Evaluation of adherence | **General questions** | **Specific questions** | **Data sources to answer the questions** |
| --- | --- | --- | --- |
| Content | To what extent was each of the intervention components implemented as planned? | To what extent were the active ingredients (geriatric assessment, contact between emergency care and case manager, contact between case manager and older people and their relatives, care planning at home, continuous contact between case manager and older people) implemented as planned? | Community project leader’s diary of overall work processes and case manager’s diary of each participant.  Observations of case manager’s work practices according to an observation protocol.  Regular interviews with the nurse with geriatric expertise, the case manager and the multi-professional team. |
| Frequency/Duration (Dosage, Dose delivery) | Was the intervention implemented as often and as long as planned? | To what extent was a quick geriatric assessment done?, To what extent were hospital wards contacted by the case manager within a couple of days after patient hospitalization?, To what extent was the care planning done early after homecoming from hospital?, To what extent were the relatives offered an early contact?, To what extent were the care efforts put in place soon after the care planning?, To what extent did the case manager made a follow-up call to patients within one week after care planning?, To what extent did the case manager have contact with patients once a month (or more often)? | Community project leader’s diary of overall work processes and case managers diary of each participant.  Observations of case manager’s work practices.  Regular interviews with the nurses with geriatric expertise, case manager and multi-professional team. |
| Coverage  (Reach) | What proportion of target group participated in the intervention? | To what extent did the older persons contact other care providers than the case manager (for instance primary care) ? | Interviews with older persons |
| 2. Potential moderating factors |  |  |  |
| Participant responsiveness  (Dose received) | How did the participants get engaged with the intervention services?  How satisfied were the participants with the intervention services?  How did the participants perceive the outcomes and relevance of the intervention? | To what extent did the older persons contact the case manager?, To what extent did the relatives participate to the support program?, How satisfied were the older persons and their relatives with the intervention services? | Questionnaire items to the older persons in the 6- and 12-month and 2-year follow-up measurements.  Interviews with a selection of older persons (approx. 10).  Questionnaire survey to relatives. |
| Intervention complexity | How complex is the intervention? |  | A group of external researchers will evaluate the intervention complexity |
| Comprehensiveness of policy description | How specific is the interventions description? |  | A group of external researchers will evaluate the comprehensiveness of policy description. |
| Strategies to facilitate implementation | What strategies were used to support implementation?  How were these strategies perceived by staff involved in the project? |  | Interviews with all project leaders, (research, community, and hospital)  Regular interviews with the nurses with geriatric expertise, case manager, multi-professional team, and other relevant actors. |
| Quality of delivery | How was the quality of delivering the intervention components? |  | Regular interviews with the nurses with geriatric expertise, case manager, and multi-professional team.  Interviews with older persons |
| Recruitment | What recruitment procedures were used to attract individuals to the intervention?  What constituted barriers to maintaining involvement of individuals? | What information did the geriatric nurses give to the patients when recruiting participants?,  Were there some barriers at the hospital or at the community organizations regarding maintaining continued involvement? | Regular interviews with the nurses with geriatric expertise, case manager and multi-professional team. |
| Context | What factors at political, economical, organizational and work group level affected the implementation? | Did any political, economical or organizational changes effect the intervention implementation? How did leaders and colleagues in the organization response to the intervention? | Investigation of relevant documents, for instance notes from steering group meetings.  Community project leader’s diary and case manager’s diary.  Observations of case manager.  Regular interviews with the nurses with geriatric expertise, case manager, multi-professional team and other relevant actors. |
