# Supplementary material for: Systematic evaluation of implementation fidelity of complex interventions in health and social care
Source: Implement Sci. 2010 Sep 3;5:67. doi: 10.1186/1748-5908-5-67 (PMC2942793; doi:10.1186/1748-5908-5-67)
Supplement: Additional file 2 — A process-evaluation plan for Palliative care in community older people nursing homes - support for nursing staff. [file 1748-5908-5-67-S2.DOC]

**A process-evaluation plan for a *palliative care in community older people nursing homes – support for nursing staff* intervention**

**Program description and logic model**

The purpose of the project is to develop, implement, and evaluate a support program for palliative care for nursing staff and workplace leaders in nursing facilities for older people. The support program is based on a study circle model that combines participants’ reflections and discussions based on their expertise and work experiences. The model includes having participants learn to question their work practices and develop new ways of working and solving problems at work. The program consists of regular meetings for each professional group and cross-professional workshops. Reading materials regarding palliative care and other related relevant subjects will be provided prior to each study circle. An external facilitator will lead the circles.

Participants are expected to get direct support in terms of improved expertise as well as indirect support in terms of a collective platform for reflections, discussion, stimulation, and concrete improvement work. The theoretical framework for the study is that support in terms of expertise development and coaching positively affects staff attitudes towards care recipients, their work satisfaction, and general wellbeing. Staff competence and attitudes are seen as key aspects for how care recipients and their relatives experience the care provided. Leadership is seen as central when implementing a support program for staff members, and therefore workplace leaders will be offered their own study circle programs.

The impact of the support program is measured in staff work satisfaction, feelings of safety at work, quality of care, care recipients’ care consumption, relatives’ perceptions of care quality, and feelings of safety.

Table 1. Logic model

| **Core inputs** | **Immediate impacts** | **Short-term impacts** | **Behavioral and other outcomes** |
| --- | --- | --- | --- |
| Study circles for nurses’ aides, registered nurses,  and workplace leaders with a facilitator.  Cross-professional workshops with a facilitator. | Increased knowledge regarding palliative care.  Possibility to reflect and question work practices.  Leaders: Possibility to reflect and question own leadership activities.  Support from the colleagues, facilitator, and workplace leader. | Changed attitudes towards care recipients.  Increased feelings of safety at work.  Improved work satisfaction.  Ideas for improvement of work practices. | Concrete improvement plans and guidelines to develop work practices.  Improved quality of care.  Decreased use of health care resources among care recipients.  Higher satisfaction with care and feelings of safety among relatives/families. |

**Complete and acceptable delivery**

The ideally implemented palliative support intervention will consist of the following components and stages:

- External facilitators for the study circles are trained.
- A study plan will be created including information of content of each meeting.
- The study circles cover three overall themes: content of palliative care in community nursing facilities, existential aspects in caring, and contacts with relatives of care recipients in palliative care.
- Registered nurses and workplace leaders have similar themes at their meetings. In addition, they have focus on issues related to leadership.
- A total of seven study circles for nurses’ aides in each work unit.
- Each group consists of four to eight participants who come from same work unit.
- There is a total of seven study circles for registered nurses and workplace leaders.
- The study circles are organized every third week.
- Before each meeting, the participants are offered an opportunity to study the preparatory reading materials about palliative care.
- At each study circle, the reading materials are reflected upon and discussed.
- Participants’ personal experiences are discussed.
- After finishing each of the overall three study circle theme, a cross-professional workshop is organized for nurses’ aides, registered nurses, and workplace leaders.
- At the workshop the focus is on finding ways to implement improvement ideas from the study circles. This could imply measures like development of guidelines or action plans.

The control nursing facilities receive the regular activities that are provided to staff and leaders within the context of community care. The intervention project involves nurses’ aides in terms of answering to one baseline and three follow-up questionnaires on their work satisfaction, work climate, and quality of care. No other activities are offered to the control facilities from the research project.

**Process-evaluation questions and methods for answering the questions**

Table below reports questions for the process evaluation.

| **Areas to measure**  **1.** Evaluation of adherence | **General questions** | **Specific questions** | **Methods for answering questions** |
| --- | --- | --- | --- |
| Content | To what extent was each of the intervention components implemented as planned? | To what extent were the study circles and workshops implemented as planned (i.e. in accordance to study plans, including preparatory reading, reflections and discussion)? | Facilitators’ logbooks  Interviews with the facilitators  Observations of a selection of study circles and workshops  Interviews with participants |
| Frequency/Duration (Dosage, Dose delivery) | Was the intervention implemented as often and as long as planned? | Were a total of eight study circles organized for each group? Were the study circles organized approx. every third week? Were there 6-8 persons in each group? Was a workshop organized after each theme in the study circles? | Facilitators’ logbooks  Interviews with facilitators  Observations of a selection of study circles and workshops  Interviews with participants |
| Coverage  (Reach) | What proportion of target group participated in the intervention? | Did a proportion of nurses’ aides, registered nurses, and leaders in the intervention organizations participate in the intervention? | Interviews with workplace leaders, facilitators, and project coordinators |
| 2. Potential moderating factors |  |  |  |
| Participant responsiveness  (Dose received) | How did the participants become engaged with the intervention services?  How satisfied were the participants with the intervention services?  How did the participants perceive the outcomes and relevance of the intervention? | To what extent did nurses’ aides, nurses, and leaders read the material provided, attend the study circles and workshops, actively reflect and discuss?  How satisfied were nurses’ aides, registered nurses, and leaders with the intervention services? | Questionnaire items to nurses’ aides in the follow-up questionnaires  Interviews with nurses’ aides, registered nurses, and leaders  Facilitators’ logbooks  Interviews with facilitators |
| Intervention complexity | How complex is the intervention? |  | A group of external researchers will evaluate the intervention complexity. |
| Comprehensiveness of policy description | How specific is the intervention description? |  | A group of external researchers will evaluate the comprehensiveness of policy description. |
| Strategies to facilitate implementation | What strategies were used to support implementation?  How were these strategies perceived by staff involved in the project? |  | Interviews with facilitators and project coordinators |
| Quality of delivery | How was the quality of delivering the intervention components? |  | Interviews with facilitators, project coordinators, nurses’ aides, registered nurses, and leaders.  Facilitators’ logbooks  Observations of a selection of study circles and workshops |
| Recruitment | What recruitment procedures were used to attract individuals to the intervention?  What constituted barriers to maintaining involvement of individuals? | Who recruited the participants and how? What information was given when recruiting participants?  What characterizes persons who did not want to participate?  Were there some barriers regarding maintaining continued involvement? | Interviews with the researcher team who selected intervention organizations  Interviews with facilitators  Interviews with nurses’ aides, registered nurses and leaders |
| Context | What factors at political, economical, organizational, and work group level affected the implementation? | What factors at political, economical, organizational, and workgroup level affected the implementation of the study circles and workshops? | Interviews with facilitators, project coordinators, nurses’ aides, registered nurses, and leaders  Facilitators’ logbooks  Relevant documentation  Questionnaire items to nurses’ aides in the follow-up questionnaires |
