# Supplementary material for: Systematic evaluation of implementation fidelity of complex interventions in health and social care
Source: Implement Sci. 2010 Sep 3;5:67. doi: 10.1186/1748-5908-5-67 (PMC2942793; doi:10.1186/1748-5908-5-67)
Supplement: Additional file 3 — A process-evaluation plan for the Supported Employment (SE) among people with severe mental illness - a randomized controlled trial. the files include detailed evaluation plans for each intervention study. [file 1748-5908-5-67-S3.DOC]

**A process-evaluation plan for the *Supported Employment (SE) among people with severe mental illness – a randomized controlled trial* intervention**

**Program description and logic model**

The purpose of Supported Employment (SE), according to the Individual Placement and Support model, is to help people with severe mental illness find and keep employment that matches their strengths and past experience. The approach includes individual support and supervision from an employment specialist in the job-seeking, job-training, and job-keeping processes. The employment specialist is the leader of the intervention, working closely with the mental health care team. Thus, the participant also has access to psychiatrists, psychologists, nurses, social workers, occupational therapists, and other care providers, who will collaborate to provide optimal support. The theoretical framework for the study is that work enables people to integrate socially, and provides them with opportunities to explore and master their environment and thereby become integrated in society. Engaging in meaningful work is expected to be related to the decrease of psychiatric symptoms, and to higher self-esteem, better quality of life, financial independence, empowerment, and self-actualization.

Table 1. Logic model of Supported Employment

| **Core inputs** | **Immediate Impacts** | **Short-Term Impacts** | **Behavioral and Health Outcomes** |
| --- | --- | --- | --- |
| - Contact  with an employment specialist | - vocational profile including analyses of participant’s skills, preferences, and experience.  - individualized help to seek job  - support when meeting employers and when working | - higher level of engagement and community participation  - obtaining employment  - higher income | - improved empowerment, job-readiness, self-image, and quality of life |

**Complete and acceptable delivery**

The most standardized SE approach, the Individual Placement and Support Model (IPS) [1], is used in this study. The essence of IPS is a direct, individualized search for competitive employment which in general does not involve prolonged pre-employment preparation, counseling, and training [2]. IPS programs actively facilitate job acquisition and provide ongoing support once the client is employed [3]. IPS follows seven principles: (1) focus on competitive employment outcomes, (2) availability to anyone who wants to work, (3) rapidity of job search, (4) attention to client preferences in services and job searches, (5) individualized and long-term support, (6) close cooperation between employment specialists and psychiatric treatment team, and (7) personalized counseling for clients on Social Security and other benefits [4].

The following steps are taken in a complete and acceptable delivery of the intervention:

Job-seeking process (a planning process):

- Employment specialist and participant make an assessment of participant’s skills, abilities, strengths, weaknesses, lifestyle, resources, disabilities, vocational history, and preferences and capabilities for work.
- Employment specialist and participant create a vocational profile of issues related to employment.
- Employment specialist provides a participant with opportunities to actively select a job compatible with participant’s interest, aspirations, needs, background experience, and lifestyle conditions.

Job-finding process:

- Job-finding begins as soon as possible after the initial assessment.
- A participant is helped with tasks like compiling a CV , responding to any advertisements for work, writing letters to places of work, and inquiring directly about jobs.
- The finding process is individualized according to the strengths, preferences, and experience of the participant.
- Employment specialist uses his/her network to identify work opportunities.
- Participant has control and information enabling choices.
- Support from mental health team and participant’s family is important.

Meeting a potential employer:

- Employment specialist helps to arrange a meeting between participant and employer and participates in that if participant so desires.
- Issues to discuss include hours of work, terms and conditions of employment, workplace culture, support required by a participant, support available from an employment specialist and co-workers, health and safety requirements.

Ongoing support:

- If employment is obtained, ongoing support from the employment specialist is offered.
- The support is individualized according to the preferences of the participant.
- The support is planned and evaluated by the participant, employer, and employment specialist.
- Support is offered as long as the participant wants it.

**Process-evaluation questions and data collection methods to answer the questions**

Table 2, below, lists questions for the process evaluation.

| **Areas to measure**  **1.** Evaluation of adherence | **General questions** | **Specific questions** | **Question-answering methods** |
| --- | --- | --- | --- |
| Content | To what extent was each of the intervention components implemented as planned? | To what extent was each of the intervention components within the four stages of the SE program implemented as planned? | Will be measured by IPS fidelity scale |
| Frequency/Duration (Dosage, Dose delivery) | Was the intervention implemented as often and as long as planned? | To what extent was the job-finding process started “as soon as possible” after the initial assessment? To what extent is “ongoing support” offered to participants? To what extent was the support offered as long as the participant needed it? | Will be measured by IPS fidelity scale |
| Coverage  (Reach) | What portion of target group participated in the intervention? | Who decided to participate?  What characterized those who participated and those who did not want to participate? | Interviews with recruiters (participants’ case managers and project leader) |
| 2. Potential moderating factors |  |  |  |
| Dose received  (Participant responsiveness) | How did the participants get introduced to the intervention services?  How satisfied were the participants with the intervention services?  How did the participants perceive the outcomes and relevance of the intervention? | To what extent did participants contact the employment specialists?  To what extent did participants actively participate in the sessions with the coaches?  To what extent did participants use materials or recommendations from the intervention?  How satisfied were the participants with the intervention services? | Questions to the participants in the 6- and 18-month follow-up measurements.  Interviews with employment specialists. |
| Intervention complexity | How complex is the intervention? |  | A group of external researchers will evaluate the intervention complexity. |
| Comprehensiveness of policy description | How specific is the intervention description? |  | A group of external researchers will evaluate the comprehensiveness of policy description. |
| Strategies to facilitate implementation | What strategies were used to support implementation?  How were these strategies perceived by staff involved in the project? |  | Interviews with employment specialists. |
| Quality of delivery | How was the quality of delivering the intervention components? |  | Interviews with employment specialists. |
| Recruitment | What recruitment procedures were used to attract individuals to the intervention?  What constituted barriers to maintaining involvement of individuals? | Who recruited the participants and how? What information was given when recruiting participants?  What characterizes persons who did not want to participate?  Were there some barriers regarding maintaining continued involvement? | Interviews with recruiters (participants’ case managers and project leader).  Investigations of relevant documents.  Interviews with employment specialists. |
| Context | What factors at political, economical, organizational, and work group level affected the implementation? | How did organizations and persons outside the intervention respond to the intervention? Did any political, organizational, or economical changes affect the implementation? | Interviews with employment specialists and relevant persons outside the intervention (for instance, municipal authorities, mental health care staff, employers, social insurance, and public employment offices)  Relevant documents, for instance notes from meetings. |

1. Drake RE, Becker DR, Clark RE, Mueser KT: **Research on the individual placement and support model of supported employment.** *Psychiatric Quarterly* 1999, **70:**289-301.

2. Rinaldi M, Perkins D: **Implementing evidence-based supported employment.** *Psychiatric Bulletin* 2007, **31:**244-249.

3. Bond, Becker DR, Drake RE, Rapp CA, Meisler N, Lehman AF, Bell MD, Blyler CR: **Implementing Supported Employment as an evidence-based practice.** *Psychiatric Services* 2001, **52:**313.

4. Burns T, Catty J, Becker T, Drake RE, Fioritti A, Knapp M, Lauber C, Rössler W, Tomov T, Van Busschbach J: **The effectiveness of supported employment for people with severe mental illness: a randomised controlled trial.** *The Lancet* 2007, **370:**1146-1152.
